# Bioactivity and biochemical efficacy of chitinase and *Justicia brandegeana* extract against Red Palm Weevil *Rhynchophorus ferrugineus* Olivier (Coleoptera: Curculionidae)

**DOI:** 10.1002/fsn3.1787

**Published:** 2020-07-16

**Authors:** Ayman A. Shehawy, Magda T. Ibrahim, Enas S. Aboutaleb, Sameer H. Qari

**Affiliations:** ^1^ Biology Department Aljumum University College Umm Al‐Qura University Makkah Saudi Arabia; ^2^ Plant Protection Research Institute Agricultural Research Centre Giza Egypt; ^3^ Pharmacognosy Department Faculty of Pharmacy Sinai University Cairo Egypt; ^4^ Pharmacognosy Department Faculty of Pharmacy Al‐Azhar University Cairo Egypt

**Keywords:** biochemical, chitinase, *Justicia brandegeana*, Red Palm Weevil

## Abstract

The red palm weevil *Rhynchophorus ferrugineus* is a large polyphagous insect, and this study was carried out to isolate chitinase from *Beauveria bassiana* as well as phytochemical screening of *Justicia brandegeana* to elucidate its effect as biocontrol agents against the red palm weevil and its possible effect on enzymatic bioactivity. It is the first time that the lipoid constituents of *J. brandegeana* were examined by both gas–liquid chromatography (GLC) and gas chromatography–mass spectrometry (GC/Mass). The results showed that the highest rates of mortality in treated prepupae were 35.0% and 30.0% with the higher concentration of chitinase (25 ppm) and petroleum ether extract of *J. brandegeana* (1,200 ppm), respectively. Moreover, changes in enzyme activity of ALP, PO, GPT, and GOT in the prepupal stage after treatment with LC_50_ chitinase and *J. brandegeana* extract were 36.63 & 14.32, −21.99 & 41.20, −11.02 & 47.05, and −36.00 & 21.43% compared with untreated control, respectively. This study demonstrated effectiveness of chitinase, and the petroleum ether extract of *J. brandegeana* has potent effect against *Rh. ferrugineus* due to its disturbance effect on the enzymatic system, protein as well as DNA damage.

## Graphical abstract

Chitinase was extracted from *Beauveria bassiana* and Phytochemical screening of *Justicia brandegeana* was performed to elucidate its effect as biocontrol agents against *Rhynchophorus ferrugineus* and its possible effect on enzymatic bioactivity of ALP, PO, GPT, and GOT as well as on DNA and protein content. This study demonstrated effectiveness of chitinase, and the petroleum ether extract of *J. brandegeana* has potent effect against *Rh. ferrugineus* due to its disturbance effect on the enzymatic system, protein as well as DNA damage.

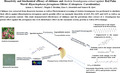

## 1 INTRODUCTION

*Rhynchophorus ferrugineus* Olivier (Coleoptera: Curculionidae) that commonly named the red palm weevil (RPW) is a large polyphagous insect native to several states including southern Asia and Melanesia. Also, it is one of the most important pests of several palm species. Study of Barranco, De la pea, Martin, and Cabello (2000) was reported that it invades more than 21 palm species worldwide, including date palm (Giblin‐davis, 2001) and coconuts palms (Faleiro, 2006). Nowadays, the RPW is widely found in Africa, Europe, Asia, and Oceania (Yuezhong, Zeng‐rong, Ruiting, & Lian‐Sheng, 2009). On the other hand, the climatic conditions of the date palm‐growing countries and the intensive modern date palm farming practices, helping in the establishment of this pest (Faleiro, 2006). Nowadays, the biological control including entomopathogenic fungi and natural plant extracts is one of the important tools in *Rh. ferrugineus* control (Mazza et al., 2014). Also, it was found that, the contamination of larval instars with *Beauveria bassiana* spores resulted in 50%–100% larval mortality, this mortality effect may due to chitinase secretion which broke down the main component of the cuticle chitin to low molecular weight of soluble and insoluble oligosaccharides (Glare, Placer, Nelson, & Reay, 2002; Schwarz & Moussian, 2007).

Chitinases are the enzymes that breaking down glycosidic bonds in chitin; hydrolytic enzyme (Jollès & Muzzarelli, 1999). Thus, chitinobiosidases cleave diacetylchitobiose units from nonreducing end of the chitin chain and disaccharides released. While, endochitinases cleave glycosidic linkages along the chitin chain producing low molecular mass of oligomers; chitotrioses and diacetylchitobioses (Guthrie, Khalif, & Castle, 2005). Whereas, chitinases of Eukaryotes, fungal chitinase, plant chitinase, and endo‐beta‐N‐acetylglucosaminidases sharing weak amino acid sequence at the certain regions of each enzyme. These regions may assume to be important for catalytic activities of the enzymes (Watanabe et al., 1993). Therefore, previous research by Mubarik, Mahagiani, Anindyaputri, Santoso, and Rusmana (2010) showed that the chitinase of some fungus strain showed their ability to degrade exoskeleton chitin of the whitefly. Also, it can be used for controlling leaf miner such as *aphis gossypii*. Moreover, study of Moussa, Shehawy, Baiomy, Taha, and Ahmed (2014) was isolated chitinase protein of 55kD from *B. bassiana* and concluded for partially extraction and purification of chitinase enzyme which had a significant potency in two aphid species compared with chemical insecticide Pirimicarb.

Recently, plant extracts used widely in pest control as a safe tool to the environment. Lignans from *Justicia. flava* were reported to cause ataxia, decrease in motor activity, and decreases muscular tone (Navarro, Alonso, & Navarro, 2011). Furthermore, the elenoside which isolated from leaves of *Justicia hyssopifolia* showed decrease in the muscular tone at doses of 25, 50, and 100 mg/kg in mice. *Justicia glauca* extract has insect antifeedant and insecticidal effect (Gayatri, Srinivasulu, & Hemalatha, 2018). Moreover, *J. hyssopifolia* extract has cytotoxic activity (Kavitha, Sridevi‐Sangeetha, Sujatha, & Umamaheswari, 2014).

On the other hand, many enzymes involved in detoxification; alkaline phosphatase (ALP) is the common hydrolytic enzyme, which play an important role in hydrolyzation of phosphomonoesters under alkaline conditions. ALP is used as enzyme marker for brush border membrane (Wolfersberger, 1984) and is indicator for tissue cytolysis during the insect development (Dadd, 1970). *Peganum harmala, Senna alexandrina* extract showed an increase in Alkaline phosphatase against *Aphis craccivora* compared with the control (Shehawy, Khalil, Maklad, & Qari, 2019). Glutamine pyruvic transaminase (GPT) and glutamic oxaloacetic transaminase (GOT) are known as alanine transaminase (ALT) aspartic transferase (AST), respectively. The transaminases are the key enzymes in the process of gluconeogenesis, nonessential amino acids formation, nitrogen compound metabolism, and mainly associated with protein metabolism (Mordue & Goldworthy, 1973). Phenol oxidase (PO) convert phenols to quinones that subsequently polymerize to form melanin in melanogenesis (Söderhäll & Cerenius, 1998). Strengthening of the immune system of insects occurred by increased PO activity to challenge xenobiotics and healing, and phenol oxidase is an important tool against numerous pathogens (Cerenius & Söderhäll, 2004). During the infection of desert locust by *Metarhizium anisopliae,* the activity of the enzyme phenoloxidase decreased (Gillespie, Burnett, & Charnley, 2000). Clarifying the role of PO in the immune response of insects to fungi is important in the efficient use of entomopathogenic fungi as biocontrol agents (González‐Santoyo & Córdoba‐Aguilar, 2012).

Accordingly, the current study aimed to isolate chitinase from *B. bassiana* as well as Phytochemical Screening of *J. brandegeana* to elucidate its effect as biocontrol agents against *Rh. ferrugineus* (RPW) and its possible effect on enzymatic bioactivity of ALP, PO, GPT, and GOT as well as on DNA and protein content.

## 2 MATERIALS AND METHODS

### 2.1 Fungal growth and chitinase extraction

For chitinase preparation, *Beauveria bassiana* strain brought from central laboratory, Aljumum University College, Umm Al‐Qura University, Saudi Arabia. *B. bassiana* inoculated into Soluble‐Yeast Extract (SYG, pH 6.0) medium and then held on shaking incubator at 150 rpm and 25 ± 1°C for three days. Finally, the chitinase precipitate was collected according to salting out method described by Kim and Je (2010); broth culture centrifuged at 16,000 *g* at 4°C for 10 min. After that, the supernatant incorporated with ammonium sulfate crystals 70% (w/v) with stirring until saturation then held overnight at 4°C. and then centrifuged at 16,000 *g* at 4°C for 10 min. Finally, pellet (chitinase) was dissolved in buffer (0.1 M citrate‐phosphate (pH 6)) then filtrated into 0.2 μm sterile filter to ensure its purity.

### 2.2 Chitinase determination

In order to determine the concentration and molecular weight of chitinase enzyme, the method described by Kim and Je (2010) was performed. The concentration of the protein was determined as described by (Bradford, 1976) using BioRad assay, using Bovine Serum Albumin (BSA) as a standard. The absorbance was registered on UNICO UV‐2000 spectrophotometer at 595 nm. After that, the concentration was calculated, then stock solution was stored at −20°C for bioassay experiments.

### 2.3 Plant materials

Leaves of *Justicia brandegeana*. were collected from Orman garden in Giza Governorate, Egypt. Authenticated by Professor Dr. Soad Abdalla Hassan Professor of Taxonomy, Faculty of Science, Ain Shams University. The leaves of the plants were dried at room temperature (28–30°C) for two weeks and reduced to No.36 powder and kept in tightly closed container. Voucher specimens (J b‐1‐2013) are kept in Department of Pharmacognosy, Faculty of Pharmacy, Al‐Azhar University.

### 2.4 Preliminary phytochemical screening

The preliminary phytochemical screening on methanol leaf extracts of *J. brandegeana* which was done according to Geone and Antônio (2012) to determine the presence of volatiles, carbohydrates or glycosides (Molisch, 1986), coumarin (Van‐Dan, Kleuver, & Heus, 1960), tannins saponins and anthracene (Gupta, 1994), and flavonoids (Geissman, 1962), sterols and/or triterpenes, and alkaloids (Vogel, 1961). Also, phenolic compounds were screened according to Mabry, Markhan, and Thomas (1970). As well as phenolic compounds were screened by 2D‐PC using BAW(*n*‐butanol: acetic acid: water 4:1:5 upper layer) for the first run and 15% aqueous acetic acid for the second run with use of FeCl_3_ and Naturstoff spray reagents for the detection of polyphenols spots (Mabry et al., 1970).

### 2.5 Preparation of the petroleum ether extract (lipoidal matter)

The air‐dried powder (500 g) of leaves of the plant under investigation was extracted with petroleum ether (60–80°C) in continuous extraction apparatus. The extract was evaporated to dry under vacuum and weighed (7.25 g).

### 2.6 Fractionation of lipoid matter extract

0.2 g of the extract was taken, and 40 ml KOH 20% was added; the sample was saponified over night at room temperature and then extracted with ether (petroleum ether 40–60°C) in to two layers as follows:

1. Saponifiable matter (ether extract): add few ml of MeOH, then add few ml H_2_SO_4_ to get red color, then add few ml of petroleum ether, then washed the petroleum ether extract (H_2_O‐NaCl 10%), then dried over anhydrous sodium sulfate, and then make methylation process by CH_2_N_2_.
2. Unsaponifiable matter washed with distilled water—NaCl 10%—then dried over anhydrous sodium sulfate.

### 2.7 Gas–Liquid Chromatography (GLC) of unsaponifiable matter

Identification of a hydrocarbon and sterols content of USM fraction of the *J. brandegeana* leaves was carried out by GLC analysis (HEWLETTPACHARD 5890) with Detector FID. Qualitative identification of the different constituents was performed by comparison of their relative retention times and those of authentic references compounds. While the quantitation was based on peak area integration, carried out using HP 6890 Series Gas Chromatograph System with an HP 5973 Mass Selective Detector in central Service Laboratory, Faculty of Agriculture, Cairo University, Giza.

### 2.8 Gas Chromatography–Mass Spectrometry (GC/Mass) of fatty acids

The qualitative identification of the different contents of *Justcia brandegeana* leaves was calculated by comparison of their relative times and mass spectra with those of authentic reference compound (fatty acid methyl ester, purity 98% by GC), and carried out by central Service Laboratory, Faculty of Agriculture, Cairo University, Giza.

### 2.9 Insect rearing

Adult stages of *Rh. ferrugineus* (Red palm weevil) were collected from infected date palm tree (Al‐Qaseem area, Saudi Arabia). Adults set in groups for mating in the laboratory in small plastic cages (30 × 50 × 50 cm) approximately, on its preferable diet which called sugarcane in order to complete life cycle and giving eggs and larvae under laboratory relative humidity (80 ± 10% R.H.) and temperature of (28 ± 2°C) on sugarcane according to Hussain, Rizwan‐ul‐Haq, Al‐Ayedh, Ahmed, and Al‐Jabr (2015).

### 2.10 Larvae exposure

Each ten prepupal larvae will be placed in plastic poxes lined with filter paper, topically treated individually with different serial doses of the plant extract (*J. brandegeana*) as well as Chitinase and transferred to boxes (one larva per plastic box 35 × 20×30 mm) compared with untreated control. The boxes incubation at 27°C in darkness for 1–2 weeks. The bioassay will be repeated ten times, and the results will be calculated.

### 2.11 Enzyme sample preparations for the determination of the ALP, PO, GPT and GOT activities

The hemolymph was collected from prepupal instar after 48‐hr treatment. 0.1 ml of hemolymph was drawn into Eppendorf containing phenol oxidase inhibitor (few milligrams) to prevent tanning, then diluted with saline solution 0.7%. In order to rupture the hemocytes, the diluted hemolymph was frozen for 20 s. After that, the hemolymph specimen was centrifuged at 3200 g for 5 min at 4°C. Then, the supernatant was used for assay directly.

### 2.12 Fat body

Fat bodies were collected from prepupal instar after 48‐hr treatment and then homogenized in a saline solution (the fat body of one insect/1 ml saline solution 0.7%) using an electric homogenizer for 2 min. Then, the homogenates were centrifuged at 4,000 rpm for 15 min at 4°C. The supernatant was frozen at −20°C until use.

For biochemical investigation, ALP activity was evaluated according to Klein, Read, and Babson (1960) and the enzyme activity was recorded using spectrophotometer at 550 nm. Phenol oxidase (PO) activity was determined using the method of Oppenoort and Welling (1976). 20 μL of samples was added to microplate wells containing 180 μL 10 mmol/L catechol. At 27°C, the reaction was measured every 1 min for an hour at 420 nm. The activity of the enzyme was measured as the absorbance change rate per min. GOT and GPT activity was measured according to Harold (1975), and the enzyme activity was recorded at 546 nm using spectrophotometer. The protein content estimation was conducted according to Qari and Shehawy (2020). Bovine serum albumin (BSA) was used as a standard. DNA was extracted as Baeshin, Qari, and Sabir (2007) DNA solutions were evaluated at 260 and 280nm by UV Spectrophotometer (V2.1.4).

### 2.13 Data analysis

For bioassays, LdP‐Line^®^ software was used to determine the median lethal concentration (LC_50_) by probity analysis. SPSS software was used in analysis of variance (ANOVA) of ALP, PO, GPT, and GOT bioactivity in prepupal instar. Comparison among means carried out using the least significant difference test at *p* < .05.

## 3 RESULTS

### 3.1 Preliminary phytochemical screening

The phytochemical screening results were illustrated in Table 1. As shown in Table 1, it can be concluded that the leaves of *J. brandegeana* contain carbohydrates, glycosides, coumarins, saponins, flavonoids, phenolics, volatile oils, and triterpenes, whereas anthraquinones was absent.

**TABLE 1:** Phytochemical Screening of *J. brandegeana* leaves

Constituents	Result
Volatiles	±
Carbohydrates and/or glycosides	+
Alkaloids and/or nitrogenous bases	+
Flavonoids	+
Tannins	+
Anthraquinones	‐
Saponins	+
Coumarins	+
Iridoids	‐
Unsaturated sterols and/or terpenes	+

Note

+, available; −, unavailable; ±, may be available or not.

### 3.2 Investigation of lipoidal matter

There is no information about investigation of lipoidal matter of *Justicia brandegeana* before, so it was deemed of interest to investigate the lipoidal content of this plant. As presented in Table 2, the total lipoidal matter separated was representing 1.45% among which 62% unsaponifiable matter and 27.5% representing the total fatty acids.

**TABLE 2:** Percentages of USM and TFA in *J. brandegeana* leaves

% lipoid matter	% composition	
1.45	USM	TFA
	62%	27.5

Abbreviations: TFA, total fatty acid; USM, unsaponifiable matter.

### 3.3 GLC of unsaponifiable matter

**FIGURE 1:**
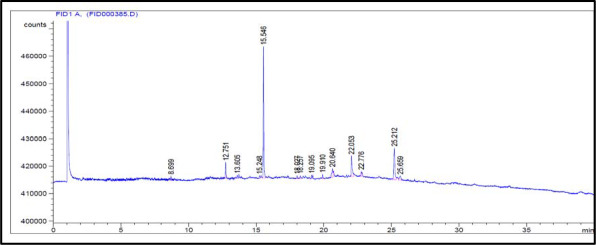
GLC total ion chromatogram of USM of *Justicia brandegeana* leaves

Data illustrated in Table 3 and Figure 1 show the relative retention time (RRT) of separated compounds as well as their relative percentages. Also, results of GLC of unsaponifiable matter in Table 3 Confirmed that the percentage of total hydrocarbon compound representing 63.311%; (Heneicosane (C_21_) is the major component (43.824%) in hydrocarbon). Whereas, the percentage of total sterol is 36.688%; (Stigmasterol is the major component (19.156%) in sterol).

**TABLE 3:** GLC of USM of *Justicia brandegeana* leaves

Peak no.	R_T_	RR_T_	Name	Area %
1	8.699	0.559	Pentadecane C15	1.23
2	12.751	0.820	Octadecane C18	5.28
3	13.605	0.875	Nonadecane C19	1.18
4	15.248	0.980	Eicosane C20	1.14
5	15.546	1.0	Heneicosane C21	43.82
6	18.027	1.159	Tricosane C23	0.90
7	18.257	1.174	Tetracosane C24	1.28
8	19.095	1.228	Pentacosane C25	1.25
9	19.910	1.280	Squalene C30	1.52
10	20.640	1.327	Octacosane C28	5.71
11	22.053	1.418	Cholesterol	11.42
12	22.776	1.465	β‐Sitosterol	2.30
13	25.212	1.621	Stigmasterol	19.16
14	25.659	1.650	Unknown	3.81

Total Hydrocarbons	63.3113%
Total Sterols	32.878%
Total unidentified	3.81

Abbreviations: C15, number of carbons is 15; Cn, number of carbon atoms; GLC, gas–liquid chromatography; RRT, retention time relative to Heneicosane RT:15.546; RT, retention time; USM, unsaponifiable matters.

### 3.4 GC/Mass of fatty acids

**FIGURE 2:**
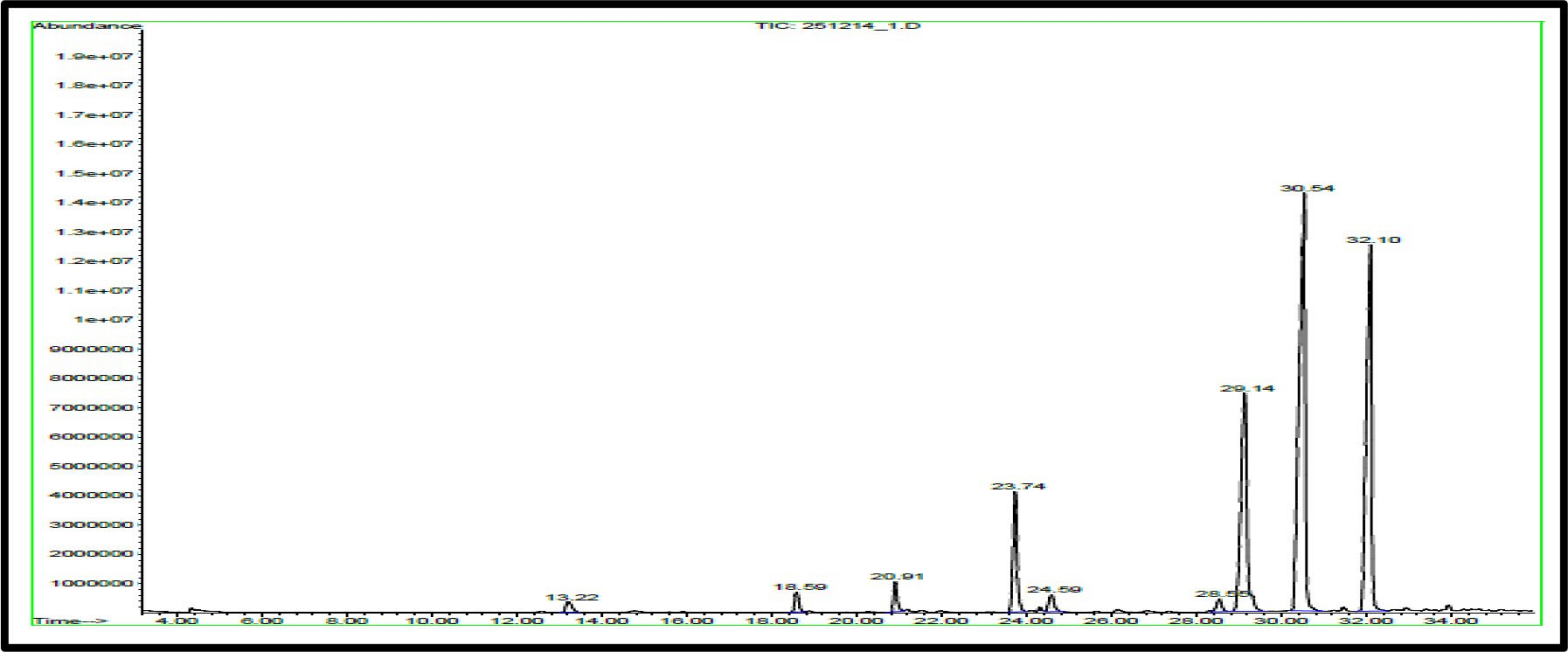
GC/MS total ion chromatogram of fatty acid of *Justcia brandegeana* leaves

**FIGURE 3:**
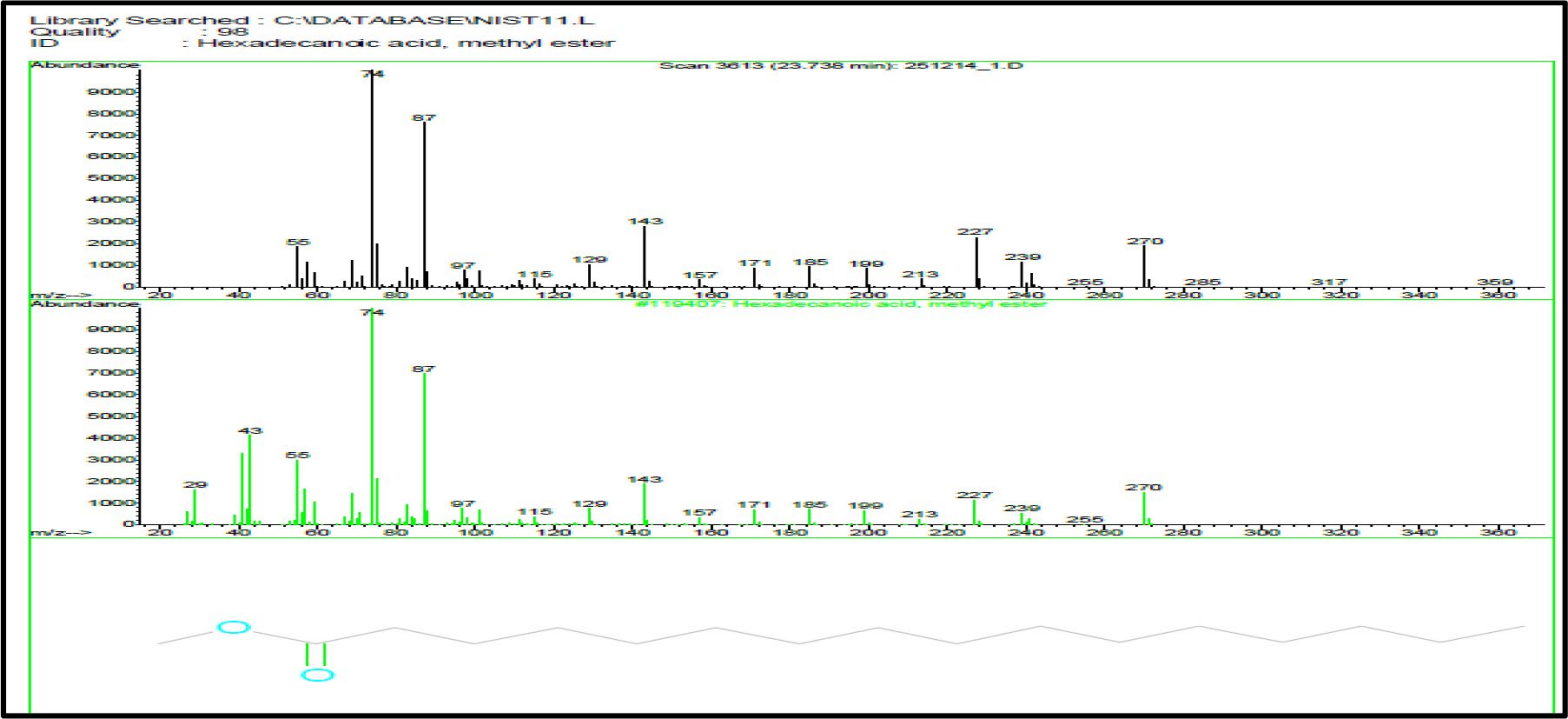
Mass spectrum of Hexadecanoic acid, ester (Palmitic acid) in *J. brandegeana* leaves

**FIGURE 4:**
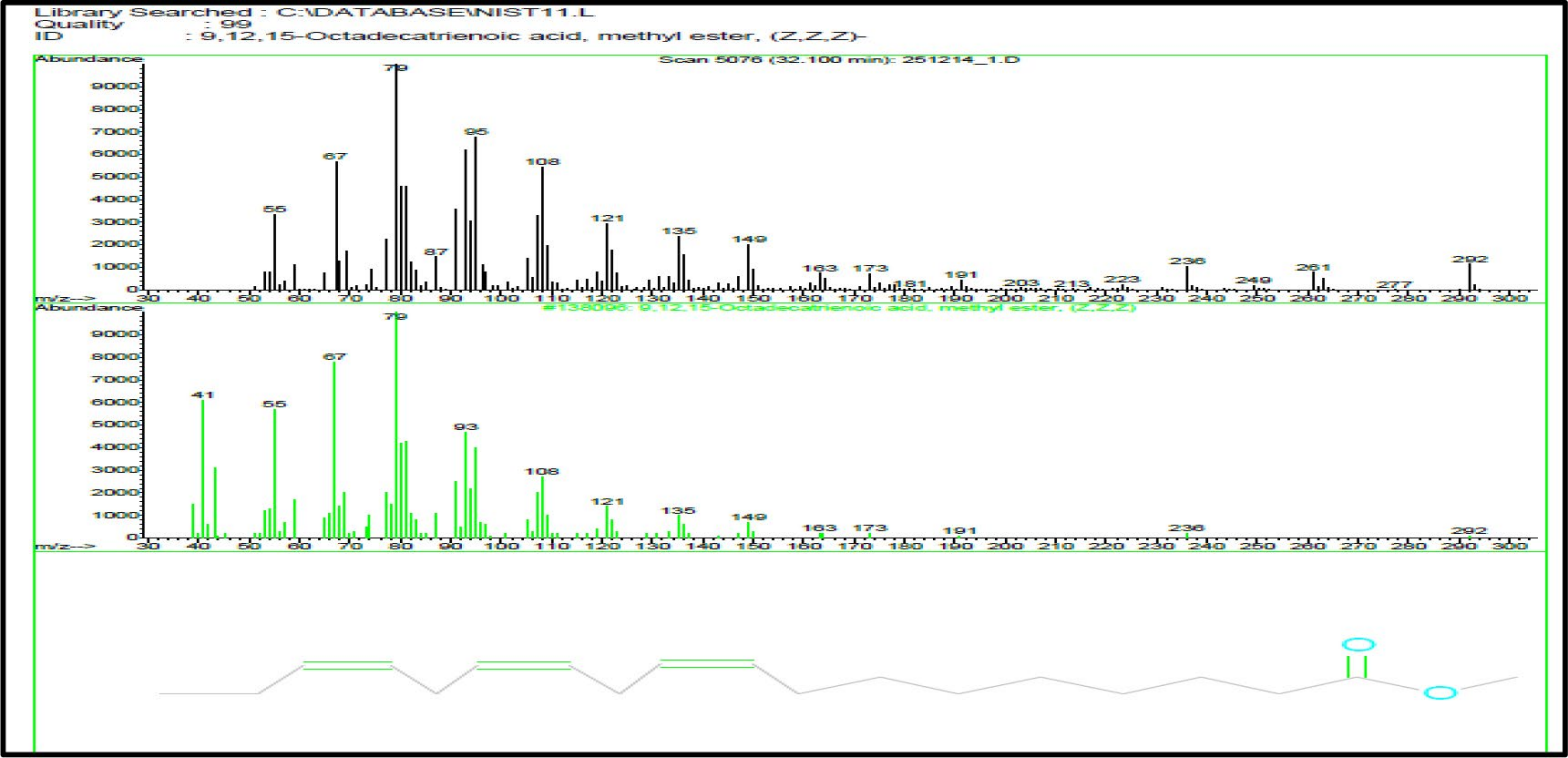
Mass spectrum of 9,12,15‐Octadecatrienoic acid, methyl ester, (Z,Z,Z)‐ in *Justcia brandegeana* leaves

Data presented in Table 4 and Figure 2 show that the percentage of identified total fatty acid in *J. brandegeana* leaves is 98.47% (Figure 3), saturated fatty acids representing 11.98% (while Palmitic acid is the major saturated one (Figure 2)), unsaturated fatty acids representing 86.49% (9,12‐Octadecadienoic acid, methyl ester (Linoleic acid; omega ‐6) is the major unsaturated component (37.98%) (Figure 4). While, unidentified compounds representing 1.53% of the oil.

**TABLE 4:** GC/Mass of fatty acid methyl esters of *Justcia brandegeana* leaves

Peak no.	R_T_	RR_T_	Name	Area %
1	13.22	0.433	Dodecanoic acid, ester (Lauric acid C12:0)	0.94
2	18.59	0.608	Methyl tetradecanoate (Myristic acid C14:0)	1.21
3	20.91	0.685	Unknown	1.53
4	23.74	0.777	Hexadecanoic acid, ester (Palmitic acid C16:0)	7.32
5	24.60	0.805	9‐Hexadecenoic acid, methyl ester, (Z)‐(Methyl palmitoleate)	1.34
6	28.55	0.935	Methyl stearate (Stearic acid C18:0)	0.98
7	29.14	0.954	9‐Octadecenoic acid, methyl ester, €‐Methyl leate	20.20
8	30.54	1.0	9,12‐Octadecadienoic acid, methyl ester Linoleic acid methyl ester (Linoleic acid C18:2)	37.98
9	32.10	1.051	9,12,15‐Octadecatrienoic acid, methyl ester, (Z,Z,Z)‐ ( Linolenic acid C18:3)	28.49

Identified saturated fatty acids %	10.45
Identified unsaturated fatty acids %	88.01
Unidentified compound%	1.53

Abbreviations: Cn, number of carbon atom; GC/Mass, Gas Chromatography–Mass Spectrometry; RRT, retention time relative to Linoleic acid RT = 30.54; RT, retention time.

### 3.5 Insect bioassay

As shown in Table 5, the lethal effect of chitinase and *J. brandegeana* extract against Date palm weevil (RPW), *Rh. ferrugineus* increased with increasing dose. Moreover, the mortality% in prepupal stage after treatment with chitinase ranged between 10% and 35% (LC_25_ = 19.6 and LC_50_ = 38.2 ppm). Whereas, the mortality% ranged between 8% and 30% in case of *J. brandegeana* extract (LC_25_ = 897.4 and LC_50_ = 3,334.46 ppm). On the other hand, the highest rates of mortality attained were 35% and 30% in treated prepupae with the higher concentration of chitinase (25 ppm) and *J. brandegeana* extract (1,200 ppm), respectively.

**TABLE 5:** Lethal effect of different concentrations of *J. brandegeana* & chitinase (ppm) applied topically onto the prepupae of the Red Palm Weevil, *Rhynchophorus ferrugineus*

Dose (ppm)		% prepupal mortality		% pupation		% pupal mortality		% adult emergence		% adult mortality		% total inhibition of adult emergence	
Control		2		98		0		98		0		2	
C	J	C	J	C	J	C	J	C	J	C	J	C	J
10	200	10	8	90	92	10	5	80	88	7	2	27	15
15	400	15	12	85	88	15	10	60	78	11	4	41	26
20	600	25	20	75	80	18	14	47	66	15	7	58	41
25	1,200	35	30	65	70	25	20	40	50	20	10	80	60

Abbreviations: C, chitinase; J, *Justcia brandegeana* extract.

Whereas the highest concentration of chitinase and *J. brandegeana* extract caused 20% and 10% mortality in the adult stages, respectively, compared with untreated control group. Meanwhile, the lower mortality% in the prepupal larvae was 10% and 8%. Whereas, treatment of adult with the lower concentration (10 & 200 ppm) of chitinase and *J. brandegeana* extract resulted in 7% and 2% mortality percentages, respectively. Generally, the total inhibition of adult emergence% reached to 80–60 after treatment with chitinase and *J. brandegeana* extract, respectively.

### 3.6 Enzyme activity

In general, it was found that the activities of ALP, PO, GPT, and GOT are greater in hemolymph than in fat body in untreated control larvae of *Rh*. *ferrugineus*.

#### 3.6.1 Alkaline phosphatase

As shown in Table 6, chitinase and the plant extract had significant effect on the ALP activity in hemolymph. Moreover, the remarkable increase in the enzyme activity was measured at LC_50_ of chitinase (change 36.63%) followed by LC_50_ of *J. brandegeana* extract (change 14.32%). Whereas, ALP in fat body exhibited variable activity by chitinase (13.1 ± 0.51, 10.7 ± 0.93 U/L, at LC_50_ and LC_20_, respectively) and plant extract (11.77 ± 1.06 and 9.68 ± 0.23 U/L, at LC_50_ and LC_20_, respectively), compared with the control (9.22 ± 0.43 U/L). However, the enzyme increased by chitinase in both tissues at the two concentrations of chitinase and the plant extract with no exception according to the concentration used.

**TABLE 6:** Alkaline Phosphatase (ALP) activity, Phenol oxidase (PO) activity, and GPT activity (U/L) in fat body and hemolymph of the *Rh*. *Ferrugineus* prepupa after 48‐hr treatment with LC_50_ and LC_25_ of Chitinase and *J. brandegeana* extract

Conc. (ppm)		Hemolymph		Fat body	
		Mean ± *SD*	Change %	Mean ± *SD*	Change %
Alkaline Phosphatase (ALP) activity (U/L)					
Chitinase	LC_50_ (38.2)	49.6 ± 7.01^a^	36.63	13.1 ± 0.51^a^	42.09
	LC_25_ (19.6)	39.4 ± 9.03^c^	8.53	10.7 ± 0.93^b^	16.05
*J. brandegeana* extract	LC_50_ (897.4)	41.5 ± 9.12^c^	14.32	11.77 ± 1.06^b^	27.65
	LC_25_ (3,334.4)	37.6 ± 7.01^d^	3.58	9.68 ± 0.23^bc^	4.98
Control	–	36.3 ± 5.49^d^	–	9.22 ± 0.43^c^	–
LSD	–	2.9	–	1.07	–
*p* value		<.002		<.001	
Phenol oxidase (PO) activity (U/L)					
Chitinase	LC_50_ (38.2)	25.22 ± 2.55^d^	−21.99	12.55 ± 0.52^e^	−28.93
	LC_25_ (19.6)	30.78 ± 8.25^c^	−4.73	15.80 ± 0.82^d^	−10.53
*J. brandegeana* extract	LC_50_ (897.4)	45.65 ± 8.55^a^	41.20	22.69 ± 2.00 ^a^	28.48
	LC_25_ (3,334.4)	38.33 ± 2.67^b^	18.55	19.81 ± 2.62^b^	12.17
Control	–	32.33 ± 11.76^b^	–	17.66 ± 0.82^c^	–
LSD	–	9.22	–	1.8	–
*p* value		<.005		<.003	

Note

Conc. concentration; mean ± *SD* followed with the same letter is not significantly different (*p* > .05), mean ± *SD* followed with the different letter is not significantly different (*p* < .05).

#### 3.6.2 Phenol oxidase (PO) activity

Moreover, as shown in Table 6, chitinase treatment at LC_50_ and LC_25_ had significant decreasing effect on Phenol oxidase (PO) activity in hemolymph and fat bodies compared with that of the control, with change rate (−21.99, −4.73%) and (−28.93, −10.53%), respectively. Whereas, *J. brandegeana* extract treatment at LC_50_ and LC_25_ had significant increasing effect on Phenol oxidase (PO) activity in hemolymph and fat bodies compared with untreated control. So, the change rate was (−41.20, 18.55%) and (28.48, 12.17%) respectively compared with untreated control larvae.

#### 3.6.3 Glutamic pyruvic transaminase (GPT)

Results illustrated in Table 7 showed that the GPT activity after treatment with chitinase was significantly affected and decreased in both of hemolymph and fat bodies according to the concentration which were used with no exception, the enzyme activity after treatment with LC_50_ and LC_25_ (75.63 ± 9.41, 82 ± 7.66 U/L) and (11.67 ± 1.43, 13.9 ± 3.32 U/L) compared with (85 ± 7.66, 14.75 ± 2.22U/L) in the control in hemolymph and fat bodies, respectively. Whereas, GPT activity after treatment with of *J. brandegeana* extract was increased in both of hemolymph and fat bodies; the change in enzyme activity was measured at LC_50_ of *J. brandegeana* extract (47.05%) in hemolymph followed by (34.03%) in fat bodies. However, the enzyme increased by *J. brandegeana* extract in both of tissues. While, it was decreased after treatment with chitinase in both of tissues (hemolymph and fat bodies).

**TABLE 7:** GPT activity (U/L), GOT activity (U/L) in fat body & hemolymph and DNA & protein content of the *Rh*. *ferrugineus* prepupa after 48‐hr treatment with LC_50_ and LC_25_ of Chitinase and *J. brandegeana* extract

Conc. (ppm)		Hemolymph		Fat body	
		mean ± *SD*	Change %	mean ± *SD*	Change %
GPT activity (U/L)					
Chitinase	LC_50_ (38.2)	75.63 ± 9.41^d^	−11.02	11.67 ± 1.43^cd^	−20.88
	LC_25_ (19.6)	82 ± 7.66^d^	−3.52	13.9 ± 3.32^c^	−5.76
*J. brandegeana* extract	LC_50_ (897.4)	125 ± 13.14^a^	47.05	19.77 ± 1.20^a^	34.03
	LC_25_ (3,334.4)	101 ± 11.11^c^	18.82	16.14 ± 1.36^b^	9.42
Control	–	85 ± 7.66^d^	–	14.75 ± 2.22c	–
LSD	–	23.37	–	2.23	–
*p* value		<.006		<.001	
GOT activity (U/L)					
Chitinase	LC_50_ (38.2)	76.86 ± 6.74^e^	−36.00	25.26 ± 2.25^e^	−33.89
	LC_25_ (19.6)	122.8 ± 11.46^c^	2.23	37.1 ± 2.14^c^	−2.90
*J. brandegeana* extract	LC_50_ (897.4)	145.86 ± 5.46^a^	21.43	45.11 ± 3.40^a^	18.05
	LC_25_ (3,334.4)	128.8 ± 12.0^c^	7.23	41.31 ± 3.17^b^	8.11
Control	–	120.11 ± 13.68^d^	–	38.21 ± 3.43^c^	–
LSD	–	8.75	–	3.1	–
*p* value		<.009		<.001	

Conc. (ppm)		Con. of DNA (ng/µl)	Total protein (mg/gb. wt.) (TP)	
		Mean ± *SD*	Mean ± *SD*	Activity ratio
Chitinase	LC_50_	188.66 ± 7.84^b^	14.3 ± 1.6^b^	0.66
	LC_25_	222.3 ± 10.66^a^	18.2 ± 1.3^a^	0.85
*J. brandegeana* extract	LC_50_	155.66 ± 7.66^c^	11.76 ± 1.6^c^	0.54
	LC_25_	198.81 ± 9.6^b^	16.41 ± 1.3^b^	0.76
Control	–	248.6 ± 12.66^a^	21.40 ± 2.3^a^	–
LSD	–	26.6	3.4	–
*p* value		<.002		<.001

Note

Conc. concentration; mean ± *SD* followed with the same letter is not significantly different (*p* > .05), mean ± *SD* followed with the different letter is not significantly different (*p* < .05), activity ration = protein in treated stage/ protein in untreated stage.

#### 3.6.4 Glutamic oxaloacetic transaminase activity (GOT)

As shown in Table 7, the GOT activity in hemolymph and fat bodies After treatment with chitinase was investigated, it was found that the enzyme activity after treatment with LC_50_ and LC_25_ was (76.86 ± 6.74, 122.8 ± 11.46 U/L) and (25.26 ± 2.25, 37.1 ± 2.14 U/L) compared with (120.11 ± 13.68, 38.21 ± 3.43 U/L) in the control in hemolymph and fat bodies, respectively. Whereas, GOT activity after treatment with of *J. brandegeana* extract was increased in both of hemolymph and fat bodies; the change in enzyme activity was measured at LC_50_ of *J. brandegeana* extract (21.43%) in hemolymph followed (18.05%) in fat bodies. Thus, the results confirmed that GOT increased after treatment with *J. brandegeana* extract in hemolymph and fat bodies. Whereas, hemolymph and fat bodies were decreased after treatment with chitinase in both of tissues.

#### 3.6.5 DNA and total protein

Results illustrated in Table 7 show the that the treatment of prepupal stage with chitinase and *J. brandegeana* extract at LC_50_ had significant decrease effect on DNA and Total Protein compared with that of the control, with activity ratio (0.66–0.54) in total protein, respectively. Whereas, in case of application of LC_25_ of chitinase and *J. brandegeana* extract against prepupal stage associated with protein activity ratio (0.76–0.85) compared with the control, respectively. Also, this decreasing in the total protein value was directly proportional to DNA content in all treatment concentration.

## 4 DISCUSSION

### 4.1 Phytochemical screening for *Justicia brandegeana*

As mentioned before the plants are the most common sources of potential compounds which may have toxic or therapeutic effect. Many phytochemicals have been shown to be active against may resistant pathogenic bacteria (Gopalakrishnan, George, & Benny, 2010). The preliminary phytochemical screening of the *Justicia brandegeana* leaves extract indicated that, the extract contains carbohydrates, glycosides, coumarins, saponins, flavonoids, phenolics, volatile oils, and triterpenes, whereas anthraquinones was absent. Thus, the preliminary screening showed the importance of the chemical study of the lipoidal constituents and the total extracts of *J. brandegeana,* selected for extensive phytochemical and biological studies. Moreover, the total lipoidal matter was contained unsaponifiable matter% more than that of the total fatty acids. Meanwhile, unsaponifiable matter contains hydrocarbon compounds percentages more than that of sterol compounds. On the other hand, the GC/Mass of Fatty acids indicated that percentages of the unsaturated fatty acids are more than saturated fatty acids, these results are in the same line with that of (Kavitha et al., 2014). Moreover, Bachheti, Pandey, Archana, and Vikas (2011) concluded that *J. gendarussa* contains stigmasterol and β‐sitosterol. Also, Gayatri et al. (2018) reported the presence of Hexadecanoic acid methyl ester in *Justicia glauca, J. wynaadensis,* and *J. adathoda*. On the other hand, Jiang, Xie, and Li (2014) reported Betaine (Proteid) in *J. brandegeana*. Also, Xiao‐hua, Yun‐chang, Juan, and De‐sheng (2014) mentioned that 9,12,15‐octadecatrien‐1‐ol present in methanolic extract of *J. brandegeana*.

### 4.2 Biological activity of chitinase and *J. brandegeana* against red palm weevil larvae

Chitinases considered as the key chitin degradation enzymes, which regulate the growth and development of the insect. These are the potential target compound for insect pest management. In the current study, it was found that chitinase has toxic effect against red palm weevil larvae by degrading the cuticle of it, and this is the first study exhibiting the results of Chitinase and *J. brandegeana* extract against red palm weevil larvae. However, the current study shows the great potential effect of chitinase and *J. brandegeana* extract against red palm weevil insect. On the other hand, the results of this study are in the same line or agree with those of previous studies recorded against various insect species, chitinases are generally found in many organisms which was needs to reshape their own chitin during molting process (Jump up Sámi et al., 2001), or dissolve and digest the chitin of insects. Generally, Chitinases are the enzymes that breaking down glycosidic bonds in chitin; hydrolytic enzyme (Jollès & Muzzarelli, 1999). Therefore, previous research by Mubarik et al. (2010) showed that the Chitinase of some sp. strain showed their ability to degrade exoskeleton chitin of the whitefly. Also, can be used for controlling leaf miner or *A*.* gossypii*. Moreover, Moussa et al. (2014) isolated Chitinase protein of 55kDa from *B. bassiana* and concluded that the partially extracted and purified chitinase had a significant potency in two aphid species compared with chemical insecticide Pirimicarb. Also, some researchers suggested that *J. glauca* extract has insecticidal activity and antifeedant activity. Meanwhile, Jiang et al. (2014) reported that methanol extract of *J. brandegeana* has Antibacterial activity. Moreover, Nonadecane (toxic normal hydrocarbon (NH)) were found in the dichloromethane‐hexane crude extract of the flesh of fish samples collected from the different districts of Bangladesh (Hossain & Salehuddin, 2008).

### 4.3 Enzymatic activity in Red Palm Weevil Larvae after treatment with of chitinase and *J. brandegeana*

Alkaline Phosphatase enzyme increased by chitinase in both tissues and plant extract with no exception according to the concentration which were used. Also, PO increase is indicator for tissues cytolysis during the insect development (Dadd, 1970). In our study, Alkaline Phosphatase activity increased, in accordance with those results that reported the enhancement of ALP activity in different pests by various plant extracts (Hasheminia, Sendim, Jahromi, & Moharramipour, 2013) who recorded increase of ALP in *Pieris rapae* larvae by *Silybium marianum* methanolic extract. Also, increase of ALP in *Schistocerca gregaria* by various extracts of *Nigella sativa* (Ghoneim, Hamadah, & El‐Hela, 2016b) and *A. aegypti* larvae (Koodalingam, Deepalakshmi, Ammu, & Rajalakshmi, 2014) was recorded.

Furthermore, our findings showed that the chitinase treatment had significant decrease effect on Phenol oxidase (PO) activity in hemolymph and fat bodies compared with that of the control, Whereas, *J. brandegeana* extract treatment had significant increase effect on Phenol oxidase (PO) activity in hemolymph and fat bodies compared with that of the control. These results agree with those of who recorded that; strengthening of the immune system of insects occurred by increased PO activity to challenge xenobiotics and healing (Chang, Rahmawaty, & Chang, 2013). Phenol oxidase is an important tool against numerous pathogens (Cerenius & Söderhäll, 2004).). During the infection of desert locust by *M. anisopliae,* the activity of the enzyme phenol oxidase decreased (Gillespie et al., 2000).

As before, Glutamic pyruvic transaminase (GPT) and Glutamic oxaloacetic transaminase activity (GOT) decreased after treatment with chitinase may be due to that the transaminases are the key enzymes in the process of gluconeogenesis, nonessential amino acids formation, nitrogen compound metabolism and mainly associated with protein metabolism problem as reported by Mordue and Goldworthy (1973). While, the increase in GPT after treatment with *J. brandegeana* extract is in agree with that of Ghoneim, Hamadah, and El‐Hela (2016a) who reported that Neemazal activated GOT and GPT and all enzymes in hemolymph and fat bodies when using *Nigella sativa* extracts against *S*. *gregaria*.

Furthermore, our finding in this study indicated that chitinase and *J. brandegeana* extract treatment at LC_50_ had significant decrease effect on DNA and Total Protein, this finding is in agreement with that of Mótyán, Tóth, and Tőzsér (2013) who reported that, the decrease in total protein was occurred due to the increase of Proteolytic enzymes which required for insecticides detoxification. Also, changes in the protein content reflect changes in synthesis and degradation balance in functional and structural protein during metamorphosis and pesticide detoxification (Ghoneim et al., 2014). Meanwhile, DNA content decreased significantly after treatment with LC_50_ of the different natural compounds against *R. dominica,* due to DNA damage by such extracts compared with the control (Qari, Abdel‐Fattah, & Shehawy, 2017).

## 5 CONCLUSION

In the current study, chitinase was extracted from *Beauveria bassiana* as well as Phytochemical Screening of *Justicia brandegeana* was performed, *J. brandegeana* extract elevated ALP, PO, GPT, and GOT in both of hemolymph and fat bodies in prepupal larval instar, this increase may due to disturbance in gene expression. Whereas, Phenol oxidase (PO), PO, GPT, and GOT were decreased after treatment with chitinase. On the other hand, chitinase and the plant extract decrease the DNA and protein content. Generally, this study demonstrated effectiveness of Chitinase and the petroleum ether extract of *J. brandegeana* against red palm weevil due to its disturbance effect on the enzymatic system, protein as well as DNA damage.

## CONFLICT OF INTEREST

The authors declare that the research was conducted in the absence of any commercial or financial relationships that could be construed as a potential conflict of interest.

## AUTHOR CONTRIBUTIONS

S.A. and Q.S. conceived and designed the experiments; A.E., I.M., and S.A. performed plant extraction, GLC, and GC/Mass; S.A. and Q.S. performed chitinase extraction, bioassay, and enzymatic analysis; S.A. performed data analysis and paper writing.
